# On the clinical relevance of using complete high-resolution HLA typing for an accurate interpretation of posttransplant immune-mediated graft outcomes

**DOI:** 10.3389/fimmu.2022.924825

**Published:** 2022-09-29

**Authors:** Maria Meneghini, Anna Perona, Elena Crespo, Frederike Bemelman, Petra Reinke, Ondrej Viklicky, Magali Giral, Eduard Palou, Alba Torija, Laura Donadeu, Edoardo Melilli, Jose Zuñiga, Anett Sefrin, Nils Lachmann, Liu Hu, Petra Hruba, Cécile Guillot-Gueguen, Sophie Brouard, Josep Grinyo, Oriol Bestard

**Affiliations:** ^1^ Kidney Transplant Unit, Nephrology Department. Vall d’Hebron University Hospital, Barcelona, Spain; ^2^ Vall d’Hebron Institut de Recerca (VHIR), Universitat Autònoma de Barcelona, Barcelona, Spain; ^3^ Department of Medicine, Barcelona University, Barcelona, Spain; ^4^ Renal Transplant Unit, Department of Internal Medicine, Amsterdam University Medical Centers, Academic Medical Center - University of Amsterdam, Amsterdam, Netherlands; ^5^ Berlin Center for Advanced Therapies (BeCAT), Berlin Institute of Health Center for Regenerative Therapies (BCRT) and Department of Nephrology and Intensive Care, Charité Universitätsmedizin Berlin, Berlin Institute of Health, Berlin, Germany; ^6^ Transplant Laboratory, Institute for Clinical and Experimental Medicine (IKEM), Prague, Czechia; ^7^ Department of Nephrology, Institute for Clinical and Experimental Medicine (IKEM), Prague, Czechia; ^8^ Nantes Université, Inserm, Centre Hospitalier Universitaire (CHU) Nantes, Centre de Recherche en Transplantation et Immunologie UMR1064, Institut de Transplantation Urologie-Néphrologie (ITUN), Nantes, France; ^9^ Histocompatibility Laboratory, Immunology Department. Hospital Clinic, Barcelona, Spain; ^10^ Kidney Transplant Unit, Nephrology Department, Bellvitge University Hospital, Barcelona, Spain; ^11^ HLA- Laboratory, Charité- Universitätsmedizin Berlin, Berlin, Germany

**Keywords:** kidney transplantation, HLA typing, donor-specific antibodies, allograft rejection, precision medicine

## Abstract

Complete and high-resolution (HR) HLA typing improves the accurate assessment of donor–recipient compatibility and pre-transplant donor-specific antibodies (DSA). However, the value of this information to identify *de novo* immune-mediated graft events and its impact on outcomes has not been assessed. In 241 donor/recipient kidney transplant pairs, DNA samples were re-evaluated for six-locus (A/B/C/DRB1/DQB1+A1/DPB1) HR HLA typing. *De novo* anti-HLA antibodies were assessed using solid-phase assays, and dnDSA were classified either (1) as per current clinical practice according to three-locus (A/B/DRB1) low-resolution (LR) typing, estimating donor HLA-C/DQ typing with frequency tables, or (2) according to complete six-locus HR typing. The impact on graft outcomes was compared between groups. According to LR HLA typing, 36 (15%) patients developed dnDSA (LR_dnDSA+). Twenty-nine out of 36 (80%) were confirmed to have dnDSA by HR typing (LR_dnDSA+/HR_dnDSA+), whereas 7 (20%) did not (LR_dnDSA+/HR_dnDSA−). Out of 49 LR_dnDSA specificities, 34 (69%) were confirmed by HR typing whereas 15 (31%) LR specificities were not confirmed. LR_dnDSA+/HR_dnDSA+ patients were at higher risk of ABMR as compared to dnDSA− and LR_dnDSA+/HR_dnDSA− (logRank < 0.001), and higher risk of death-censored graft loss (logRank = 0.001). Both LR_dnDSA+ (HR: 3.51, 95% CI = 1.25–9.85) and LR_dnDSA+/HR_dnDSA+ (HR: 4.09, 95% CI = 1.45–11.54), but not LR_dnDSA+/HR_dnDSA− independently predicted graft loss. The implementation of HR HLA typing improves the characterization of biologically relevant *de novo* anti-HLA DSA and discriminates patients with poorer graft outcomes.

## Introduction

The development of *de novo* donor-specific anti-HLA antibodies (dnDSA) after kidney transplantation has a deleterious impact on graft outcomes favoring the advent of chronic antibody-mediated rejection (ABMR) and premature graft loss (1, 2). Therefore, most transplant programs have implemented routine screening of these antibodies during posttransplant follow-up (3).

While the implementation of solid-phase assays has revolutionized the assessment of antibodies with precise HLA-antigen specificities prior to and after transplantation, these advances have not paralleled the characterization of HLA typing in clinical practice. Indeed, the most frequent donor–recipient HLA genotyping worldwide still relies on one-field (two digits) low-resolution (LR) typing by sequence-specific primer PCR (SSP) or sequence-specific oligonucleotide probes (PCR-SSOP) of HLA-A, B, and DRB1 loci. However, an important body of evidence has shown that advanced high-resolution (HR) HLA genotyping based on next-generation sequencing technologies (NGS) by moving from one field to two fields (four digits) and a more complete HLA antigen assessment of up to nine distinct HLA loci (A, B, C, DRB1-3-4-5, DQ, and DP) significantly improves the accurate characterization of the degree of donor/recipient HLA matching (4). All previous studies underscored the value of such refined donor/recipient HLA typing not only to better characterize donor/recipient compatibility and to uncover all pretransplant circulating anti-HLA antibodies with donor (HLA)-antigen specificity, but also to enable to assess the degree of donor/recipient HLA disparity at the molecular level (5–9).

However, while complete HR HLA typing has shown its clinical value in the pretransplant setting, the impact of this biological information in the management of patients after transplantation has not been assessed yet. Notably, while there is still no clear evidence on how to manage transplant patients developing dnDSA, allograft biopsies to rule out ongoing immune-mediated lesions and enhancement of the immunosuppression burden are frequently performed (10–12). Therefore, the precise identification that such *de novo* anti-HLA antibodies are indeed directed against donor (HLA)-specific antigens is highly warranted to avoid invasive procedures and unnecessary rescue immunosuppressive therapies and, furthermore, to recognize those patients with true dnDSA to establish guided therapeutic strategies.

In this study, we investigated in a large cohort of kidney transplant recipients without preformed DSA participating in two prospective randomized trials whether complete two-field HR HLA typing as compared to the commonly used A-B-DRB1 one-field LR HLA typing would have an impact on the identification of posttransplant immunological events such as dnDSA formation, development of ABMR, and risk of graft loss.

## Methods

### Study population

**Figure 1:**
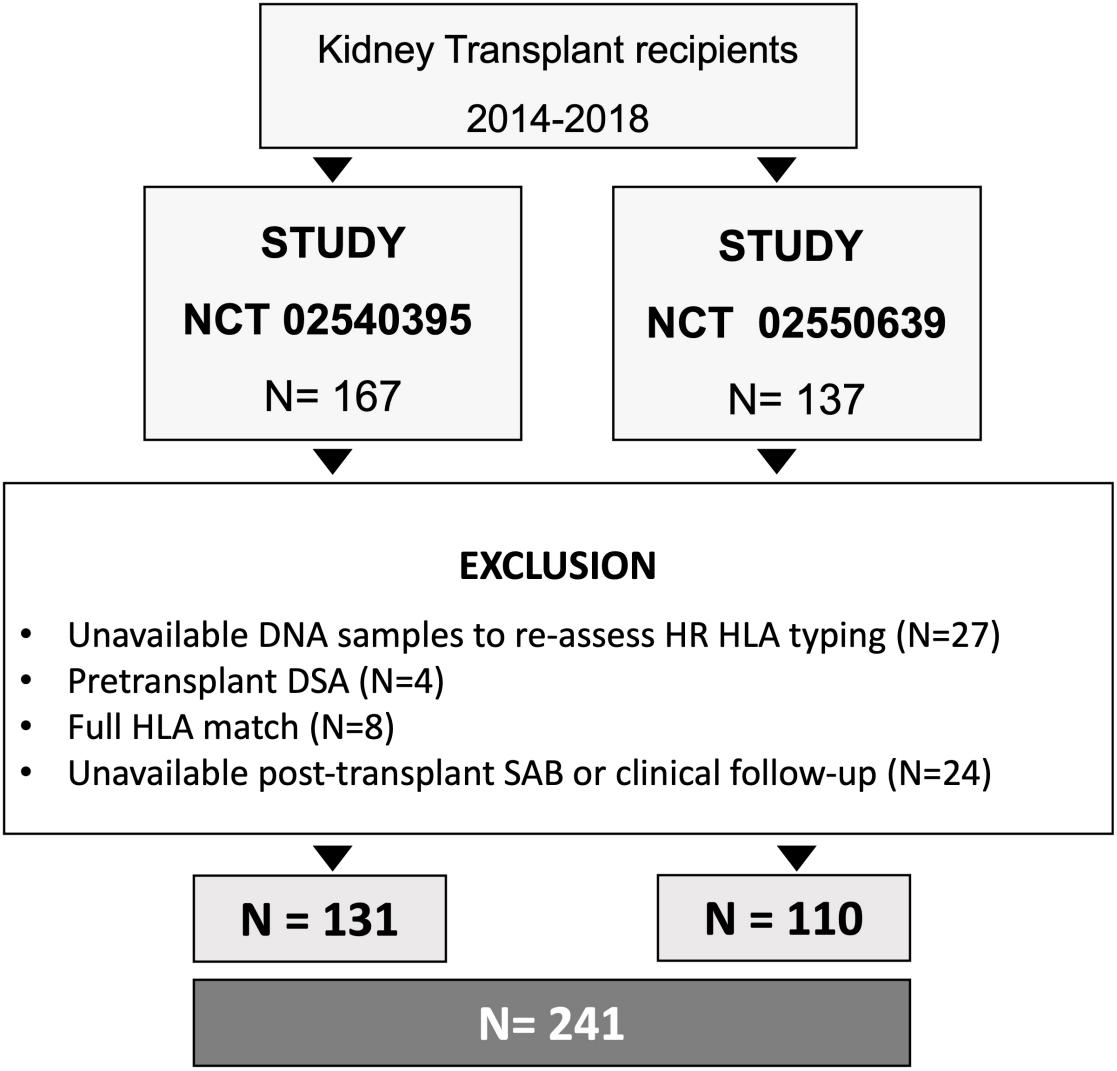
Flow chart of the study. HR, high-resolution; HLA, Human Leukocyte Antigens; DSA, donor-specific antibodies; SAB, single antigen bead assay.

Two hundred forty-one adult kidney transplant recipients from five European transplant centers (Bellvitge University Hospital, Barcelona, Spain; Amsterdam University Medical Center, Amsterdam, the Netherlands; Charités Universitätsmedizin Berlin, Germany; Institute for Clinical and Experimental Medicine-IKEM, Prague, Czech Republic; and Inserm CHU, Nantes, France), participating in two prospective randomized clinical trials (NCT 02540395 and NCT 02550639) between 2014 until 2018 and in whom donor and recipient DNA was available to perform HR HLA typing, were retrospectively investigated. All participants were ABO compatible and had a negative complement-dependent cytotoxicity crossmatch and no preformed DSA by solid-phase assay at transplantation. The absence of preformed DSA was confirmed by complete HR HLA donor typing. HLA-identical patients were excluded from the study (**Figure 1**). This study was approved by the review board of each center.

Main demographic, clinical, and immunological variables were recorded. Biopsy-proven acute rejection (BPAR) was diagnosed according to the Banff 2019 classification (13). Estimated glomerular filtration rate (eGFR) was calculated by CKD-EPI formula and allograft loss was defined as either return to chronic dialysis or re-transplantation. All clinical and immunological events were considered during the study follow-up, which was of 55 ± 15.6 months.

### HLA typing

First, recipients’ and donors’ one-field LR HLA class I (A, B) and class II (DRB1) typing were obtained using DNA-based sequence-specific primers (SSP for donors and SSOP for recipients), according to current clinical practice. As usually done in clinical practice, in case of anti-HLA antibodies against HLA-DQ and -C, donor typing at these loci was estimated using the web application of the National Marrow Donor Program (NMDP) HaploStat (http://www.haplostats.org).

In all included patients, two-field HR typing was performed using the NGS HLA typing kit on the Illumina MiSeq platform at six loci: HLA-A, -B, -C, -DRB1, -DQB1/A1, and -DPB1. Two-field donor typing at HLA-DPA1 and -DRB3/4/5 loci was performed in all cases with the presence of anti-HLA antibodies against these loci to confirm anti-donor specificity.

### Donor/recipient molecular mismatch assessment

Donor/recipient HLA eplet mismatches (EpMM) were determined by the last versions of the HLAMatchmaker software (v3.0, available at http://www.epitopes.net/downloads.html). Since two-field HLA typing is necessary to calculate EpMM, either it was predicted according to haplotype frequencies (HaploStats) from the available LR HLA typing (LR_EpMM) or using directly typed HR HLA typing was introduced in the software (HR_EpMM). Results of those two eplet MM calculations were compared.

### Detection of circulating anti-HLA antibodies

Circulating anti-HLA antibodies were assessed at baseline, at 6 and 12 months after transplantation, and yearly thereafter as well as when graft biopsies showed BPAR lesions. A single-antigen class I and class II flow beads-assay kit was used (Lifecodes, Immucor, Stanford, CA). All beads showing a normalized mean fluorescence intensity (MFI) > 500 were considered positive if (MFI/MFI lowest bead) > 5.

### Statistical analyses

All data were expressed as mean ± SD or as median and interquartile range for continuous variables, and as frequencies for categorical variables. Groups were compared using the Student’s *t*-test for normally distributed quantitative variables, and non-parametric Mann–Whitney *U*-test for non-normally distributed variables.

Bivariate correlation analyses were performed using Spearman test for non-normally distributed variables. Univariate and multivariate logistic and Cox regression models were performed to examine the factors associated with rejection and graft survival.

Kaplan–Meier probabilities of graft survival and rejection-free survival were plotted and compared using log-rank tests.

The statistical significance level was defined as two-tailed *p* < 0.05. Statistical analyses were performed with IBM SPSS Statistics (SPSS Inc., Chicago, IL) version 26.0 and GraphPad Prism version 6.0 (GraphPad, Software, La Jolla, CA).

## Results

### Study population

The main demographic and clinical characteristics of the study population are depicted in **Table 1**. Most patients were men and received a first kidney transplant, and the majority received induction treatment with anti-IL2R monoclonal antibody basiliximab.

**Table 1:** Demographic and clinical characteristics of patients included in the study.

Demographic and clinical characteristics	*n* = 241
Recipient age (years)	52.53 ± 13.79
Recipient gender (male)	164 (68)
Ethnicity (Caucasian)	228 (95)
ESRD cause	
Vascular disease	27 (11)
Diabetes mellitus	33 (14)
Glomerular disease	65 (27)
Polycystic kidney disease	47 (19)
Interstitial diseases	16 (7)
Others/unknown	53 (22)
Time on renal replacement therapy (months)	25.88 ± 40.50
Donor age (years)	53.33 ± 13.71
Donor gender (male)	119 (49)
Type of transplantation (deceased donor)	134 (56)
Kidney transplant index (first)	230 (95)
Induction immunosuppression	
rATG/anti-IL2R mAb (basiliximab)	33 (14)/208 (86)
Maintenance immunosuppression CNI-based CNI monotherapy	241 (100) 40 (16)
DGF	50 (21)
Biopsy-proven acute rejection (BPAR)	51 (21)
TCMR	34 (67)
ABMR	17 (33)
eGFR (ml/min/1.73 m^2^)	
12 months	52.97 ± 19.27
24 months	51.84 ± 19.03
36 months	49.688 ± 19.81
60 months	50.79 ± 19.15
Proteinuria (g/L)	
12 months	0.16 ± 0.35
24 months	0.19 ± 0.32
36 months	0.31 ± 0.53
60 months	0.32 ± 0.49
Death censored graft loss	16 (7)
Patient death	18 (7)

ESRD, end-stage renal disease; rATG, rabbit anti-thymoglobulin; IL2R mAb, anti-interleukin 2 receptor monoclonal antibody; CNI, calcineurin inhibitors; DGF, delayed graft function; TCMR, T-cell-mediated rejection; ABMR, antibody-mediated rejection; eGFR, estimated glomerular filtration rate.

Biopsy-proven acute rejection (BPAR) was diagnosed in 51/241 (21%) patients, 34 (67%) T-cell-mediated (TCMR) and 17 (33%) antibody-mediated (ABMR), with 8 of these ABMR (47%) showing a mixed component with concomitant TCMR lesions.

Sixteen out of 241 (7%) patients lost their graft during follow-up due to rejection (8, 50%), recurrence of primary glomerular disease (2, 12.5%), infectious complications (1, 6%), and unspecific fibrosis (5, 31%). Eighteen (7%) patients died, mainly due to cancer (7, 39%), cardiovascular (4, 22%), and infectious complications (3, 17%).

### Donor/recipient HLA antigen and molecular matching

**Figure 2:**
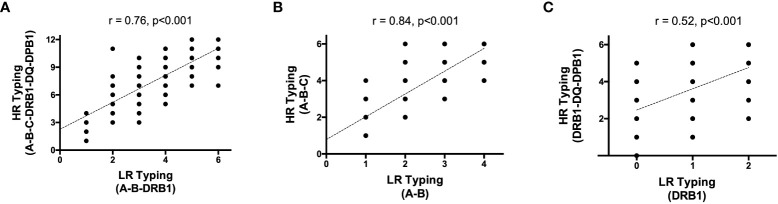
Correlation between high resolution and low resolution HLA Typing **(A)** global; **(B)** Class I and **(C)** class II. HR, high resolution; LR, low resolution.

Mean number of HLA mismatches (MM) using either LR or complete HR HLA typing is depicted in **Table 2**. According to LR HLA typing, class I (A, B) MM were 2.68 ± 0.98, class II (DRB1) MM were 1.09 ± 0.62, and the global three-locus MM were 3.78 ± 1.28. With complete and HR HLA typing, class I (A, B, and C) MM were 4.12 ± 1.42, class II (DRB1, DQA1/B1, and DPB1) MM were 3.71 ± 1.36, and the global six-locus MM were 7.79 ± 2.41. As shown in **Figure 2**, correlations between LR allelic MM and complete HR HLA MM were as follows: *r* = 0.76, *p* < 0.00 for total HLA MM; *r* = 0.84, *p* < 0.001 for class I; and *r* = 0.52, *p* < 0.001 for class II HLA MM.

**Table 2:** Mean number of HLA mismatches according to LR HLA typing (A, B, and DRB1) and HR complete HLA typing (A, B, C, DRB1, DQA/B, and DPB1).

HLA mismatches	LR HLA typing	HR HLA typing
Class I	2.68 ± 0.98	4.12 ± 1.42
Class II	1.09 ± 0.62	3.71 ± 1.36
Global	3.78 ± 1.28	7.79 ± 2.41

LR, low resolution; HR, high resolution.

Donor/recipient molecular mismatches at the eplet level (EpMM) were also assessed and calculated by introducing either haplotype frequency imputation of one-field LR HLA typing (LR_EpMM) to simulate the information available in standard clinical practice or the complete HR HLA genotyping (HR_EpMM) (**Supplementary Table 1**). The correlation between LR_EpMM and HR_EpMM for class I and class II was *r* = 0.96, *p* < 0.001 and *r* = 0.89, *p* < 0.001, respectively, and significantly lower *r* = 0.79, *p* < 0.001 when DQ and DPB1 loci are considered (**Supplementary Figure 1**).

### dnDSA identification according to different HLA typing

**Figure 3:**
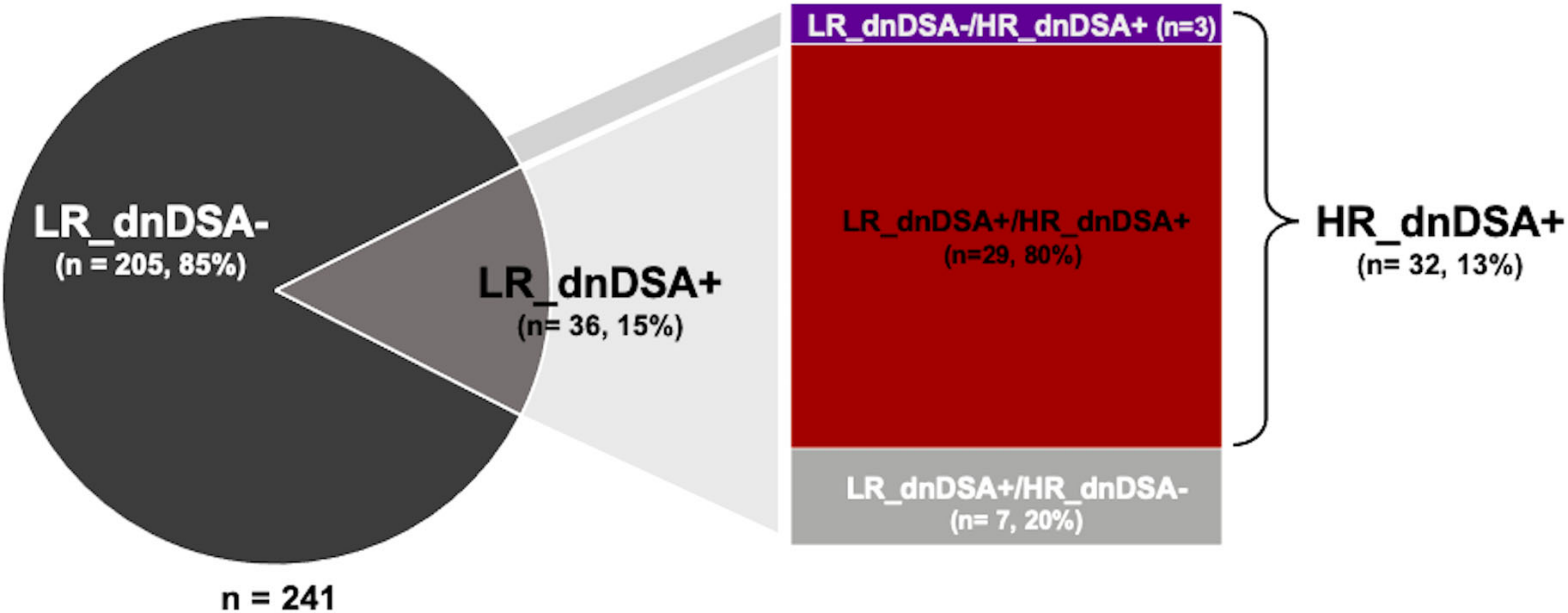
Proportion of patients with dnDSA according to LR or complete HR HLA typing. dnDSA, *de novo* donor -specific antibody; HR, high resolution; LR, low resolution.

As shown in **Figure 3**, according to LR HLA typing, 36 (15%) patients displayed any detectable dnDSA (LR_dnDSA+). In 29/36 (80%) patients, antibodies were confirmed to be DSA by complete HR HLA typing (LR_dnDSA+/HR_dnDSA+), whereas in 7 (20%) patients, these antibodies were revealed not to show anti-donor specificity using complete HR HLA typing (LR_dnDSA+/HR_dnDSA−). Furthermore, by using complete HR HLA typing, three new patients were identified to show dnDSA (LR_dnDSA−/HR_dnDSA+), anti-DP, and anti-DQ. Thus, 32/241 (13%) patients were deemed to show *true* HR_dnDSA.

Ten out of 32 (31%) patients with HR_dnDSA+ displayed anti-class I dnDSA only, 20/32 (62.5%) anti-class II only, and 2 (6.5%) patients both anti-class I and II dnDSA.

When considering the number of detected dnDSA, while 49 distinct dnDSA were detected with LR HLA typing, 34/49 (69%) were confirmed by both LR and HR HLA typing while 15 (31%) dnDSA detected by LR were not confirmed by HR HLA typing. Conversely, seven circulating anti-HLA antibodies were identified to be dnDSA only by HR HLA typing (**Table 3**).

**Table 3:** All dnDSA specificities detected by LR HLA typing and if confirmed or not by HR donor HLA typing.

	Patient no.	Anti-HLA antibody specificity	MFI range	LR donor HLA typing	HR HLA donor typing	Non-shared eplets
**LR_dnDSA+/HR_dnDSA−**	**1**	DR*11:03	1,462	Donor DRB1*11	Donor DRB1*11:04 (negative)	DRB1*11:03: Not AbV 71E
	**2**	A*11:01	9,897	Donor A*11	Donor A*11:02 (negative)	A*11:01: Not AbV:19K
	**3**	DQB1*03:03/DQA1*04:01	786	Estimated donor typing DQB1*03:03	Donor DQB1*03:03/DQA1*03:02 (negative)	DQB1*03:03/DQA1*04:01: AbV: 40GR; Not AbV: 69T 76L
	**4**	B*35:01; 35:08	2,425	Donor B*35	Donor B*35:02 (not available on SAB)	
	**5**	DQB1*03:01/DQA1*03:01; 03:02; 05:01; 06:01	1,291–2,322	Estimated donor typing DQB1*03:01	Donor DQB1*03:01/DQA1*05:05 (not available on SAB)	
	**6**	DQB1*03:01/DQA1*05:01; 06:01	18,143–14,722	Estimated donor typing DQB1*03:01	Donor DQB1*03:01/DQA1*05:05 (not available on SAB)	
	**7**	B*27:03; 27:05	1,150–1,200	Donor B*27	Donor B*27:02 (not available on SAB)	
**LR_dnDSA+/HR_dnDSA+**	**8**	B*51:01	792	Donor B*51	Donor B*51:01	
	**9**	DQB1*03:02/DQA1*02:01; 03:01; 03:02	14,825–15,601	Estimated donor typing DQB1*03:02	Donor DQB1*03:02/DQA1*02:01	
	**10**	DQB1*03:01/DQA1*03:01; 03:02; 05:01; 06:01 DQB1*06:03/DQA1*01:03	16,816–18,520 1,964	Estimated donor DQB1*03:01, DQB1*06:03	Donor DQB1*03:01/DQA1*05:01 Donor DQB1*06:03/DQA1*01:03	
	**11**	DQB1*03:02/DQA1*02:01	1,958	Estimated donor typing DQB1*03:02	Donor DQB1*03:02/DQA1*02:01	
	**12**	DQB1*03:01/DQA1*03:02; 05:01; 06:01	3,672–6,355	Estimated donor typing DQB1*03:01	Donor DQB1*03:01/DQA1*03:02	
	**13**	DQB1*02:01/DQA1*05:01	6,603	Estimated donor typing DQB1*02:01	Donor DQB1*02:01/DQA1*05:01	
	**14**	C*05:01	4,303	Estimated donor typing C*05:01	Donor C*05:01	
	**15**	A*29:01; 29:02 DQB1*03:01/DQA1*03:01; 03:02; 05:01; 06:01	2,494–3,990 7,019–13,441	Donor A*29, estimated donor typing DQB1*03:01	Donor A*29:02 Donor DQB1*03:01/DQA1*05:01	
	**16**	DQB1*03:02/DQA1*02:01; 03:01; 03:02	7,005–7,752	Estimated donor typing DQB1*03:02	Donor DQB1*03:02/DQA1*03:01	
	**17**	DQB1*05:01/DQA1*01:01	5,186	Estimated donor typing DQB1*05:01	Donor DQB1*05:01/DQA1*01:01	
	**18**	DRB1*13:01	1,966	Donor DRB1*13	Donor DRB1*13:01	
	**19**	DQB1*06:02/DQA1*01:02	18,641	Estimated donor typing DQB1*06:02	Donor DQB1*06:02/DQA1*01:02	
	**20**	B*08:01	1,871	Donor B*08	Donor B*08:01	
	**21**	A*02:01; 02:02; 02:03	1,171–2,342	Donor A*02	Donor A*02:01	
	**22**	DRB1*15:01; 15:02; 15:03	1,616–6,493	Donor DRB1*15	Donor DRB1*15:01	
	**23**	DQB1*05:01/DQA1*01:01; 01:02	1,123–1,375	Estimated donor typing DQB1*05:01	Donor DQB1*05:01/DQA1*01:01	
	**24**	B*52:01 DQ*05:01/DQA1*01:01; 01:02	1,050 1,990–2,300	Donor B*52 Estimated donor typing DQB1*05:01	Donor B*52:01 Donor DQB1*05:01/DQA1*01:01	
	**25**	A*01:01	7,900	Donor A*01	Donor A*01:01	
	**26**	C*04:01 C*06:02	1,182 1,159	Estimated donor typing C*04:01, C*06:02	Donor C*04:01 Donor C*06:02	
	**27**	B*44:03	2,000	Donor B*44	Donor B*44:03	
**LR_dnDSA−/HR_dnDSA+**	**28**	DQB1*03:01/DQA1*03:02; 05:01; 06:01	1,150–1,907	Estimated donor typing DQB1*04:02, DQB1*06:03	Donor DQB1*03:01/DQA1*06:01	
	**29**	DPB1*04:02/DPA1*01:03; 03:01	816–4,008	Not available DP typing	Donor DPB1*04:02	
	**30**	DPB1*04:01/DPA1*04:01	4,347	Not available DP typing	Donor DPB1*04:01	
**Patients with more than one possible DSA and potential misclassification**						
	**31**	DQB1*04:02/DQA1*03:01 DQB1*03:01/DQA1*03:02 DQB1*03:02/DQA1*02:01; 03:01; 03:02	2,514 2,621 6,809	Estimated donor typing DQB1*04:02, DQB1*03:02	Donor DQB1*04:02/DQA1*03:01 Donor DQB1*03:01/DQA1*03:02	
	**32**	DQB1*02:02/DQA1*02:01; 03:02; 05:01 DQB1*03:02/DQA1*02:01; 03:01; 03:02	12,406–13,055 13,991–1,512	Estimated donor typing DQB1*02:02, DQB1*03:01	Donor DQB1*02:02/DQA1*02:01 Donor DQB1*03:02/DQA1*03:01	
	**33**	B*35:08 B*51:01	1,989 1,401	Donor B*35, B*51	Donor B*35:01 (negative) Donor B*51:01	B*35:08: none
	**34**	A*02:05 DQB1*05:01/DQA1*01:01; 01:02 DPB1*02:01/DPA1*01:03 DPB1*04:02/DPA1*01:03; 03:01	2,685 2,694–7,438 8,846 13,513–14,085	Donor A*02 Estimated donor typing DQB1*05:01 DP typing not available	Donor A*02:01(negative); A*02:13 (not available on SAB) DQB1*05:01/DQA1*01:01 DPB1*02:01 DPB1*04:02	A*02:05: Not AbV 43R
	**35**	DRB1*11:01 DPB1*04:01/DPA1*01:03; 02:01	4,241 4,315–6,814	Donor DRB1*11 DP typing not available	Donor DRB1*11:02 (not available on SAB) Donor DPB1*04:01	
	**36**	A*24:02; 24:03 A*29:01 B*44:02; 44:03	4,646–10,894 2,464 5,661–5,647	Donor A*24, A*29, B*44	Donor A*24:02 Donor A*29:02 (negative) Donor B*44:03	A*29*02: none
	**37**	DRB1*03:01; 03:02 DQB1*02:01/DQA1*02:01 DQB1*03:03/DQA1*03:02; 04:01; 06:01	1,159–1,256 1,611 1,278–1,689	Donor DRB1*03 Estimated donor typing DQB1*02:01, DQB1*03:03	Donor DRB1*03:01 Donor DQB1*02:01/DQA1*05:01 (negative) Donor DQB1*03:03/DQA1*03:02	DQB1*02:01/DQA1*02:01: AbV: 47KHL
	**38**	B*57:01 DRB1*15:01; 15:02 DQB1*03:01/DQA1*03:01; 03:02; 05:01; 06:01	9,606 2,380–3,074 21,875–22,402	Donor B*57, DRB1*15 Estimated donor typing DQB1*03:01	Donor B*57:03 (not available on SAB) Donor DRB1*15:01 Donor DQB1*03:01/DQA1*05:01	
	**39**	A*01:01 B*15:12	2,000	Donor A*01, B*15	Donor A*01:01 Donor B*15:17 (negative)	B*15:12: Not AbV: 156WA

LR, low resolution; HR, high resolution; DSA, donor-specific antibody; AbV, antibody verified; SAB, single antigen bead.

In case of misclassified DSA, eplets only present on non-donor-specific HLA molecule (LR_dnDSA+) but not in the actual donor HLA molecule (HR_dnDSA−) are listed.

In red colour, misclassified dnDSA specificities not confirmed by HR HLA typing; in green, dnDSA specificities confirmed by HR HLA typing.

Most HR-confirmed anti-class II dnDSA were anti-DQ (17, 41%) dnDSA, 4 (10%) anti-DRB1, and 5 (12%) anti-DP dnDSA.

The main causes of discrepancy were different allele specificities at two-field resolution using HR typing or allele estimation (three anti-A, one anti-B, one anti-DRB1, and three anti-DQ). In other cases, discrepancies were due to absence of DQA1 (two cases) and DP (five cases) typing and missing representation of donor antigen specificities on single-antigen bead (SAB) assays in six cases (four anti-B, one anti-DRB1, and two anti-DQ) (**Table 3**).

Patients developing any confirmed dnDSA (LR_dnDSA +/HR_dnDSA+) had a trend towards higher total number of LR and HR HLA MM (LR: 4.25 ± 1.16 vs. 3.70 ± 1.28, *p* = 0.09; HR: 8.31 ± 2.02 vs. 7.71 ± 2.46, *p* = 0.06 in dnDSA+ and dnDSA−, respectively) and patients developing anti-class II dnDSA showed a higher mean number of both LR and HR class II HLA MM (LR: 1.45 ± 0.51 vs. 1.05 ± 0.62, *p* = 0.004; HR: 4.45 ± 1.14 vs. 3.63 ± 1.36, *p* = 0.009). No statistically significant difference was observed for anti-class I dnDSA and class I HLA MM (LR: 3.0 ± 1.05 vs. 2.67 ± 0.97, *p* = 0.81; HR: 4.17 ± 1.27 vs. 4.12 ± 1.43, *p* = 0.94).

### Eplet mismatch and dnDSA formation according to different HLA typing

Patients developing anti-class I dnDSA (LR_dnDSA +/HR_dnDSA+) displayed higher class I eplet MM as compared to negative patients according to LR typing (A, B) (14.83 ± 5.29 vs. 11.71 ± 5.36, in dnDSA+ and dnDSA−, respectively, *p* = 0.059) but not when considering HR A-B-C typing (14.50 ± 5.78 vs. 13.16 ± 5.95, *p* = 0.43).

Patients developing anti-class II dnDSA (LR_dnDSA +/HR_dnDSA+) showed higher class II (especially antibody-verified) eplet MM, both calculated introducing LR typing and haplotype frequency imputation of DQB1 (DRB1/DQB1 LR_EpMM: 19.32 ± 7.70 vs. 14.15 ± 10.04, *p* = 0.025; antibody-verified: 7.17 ± 3.43 vs. 5.33 ± 3.98, *p* = 0.04, in dnDSA+ and dnDSA−, respectively) and complete HR typing (DRB1/DQ/DPB1 HR_EpMM: 25.41 ± 8.89 vs. 20.49 ± 12.68, *p* = 0.019; antibody-verified: 11.09 ± 4.28 vs. 8.02 ± 5.15, *p* = 0.003) (**Supplementary Figure 2**). Similarly, DQ EpMM by haplotype frequency imputation (DQB1) and direct HR HLA typing (DQA1/B1) were significantly higher in anti-DQ dnDSA+ patients (LR_EpMM: 9.82 ± 3.5 vs. 6.10 ± 5.29 *p* = 0.003; HR_EpMM: 12.94 ± 5.09 vs. 8.49 ± 6.24 *p* = 0.002, in anti-DQ dnDSA+ and anti-DQ dnDSA−, respectively). DRB1 EpMM were not different between patients developing and not developing anti-DRB1 dnDSA.

As shown in **Table 3**, in most of the cases of LR_dnDSA+/HR_dnDSA−, only few eplets were different between the HLA molecule targeted by the circulating antibody (LR_dnDSA+) and not represented on the actual donor HLA molecules (HR_dnDSA−). In two cases, no eplets were different between the two molecules; therefore, we cannot rule out the possibility of unspecific binding, or that the antibody is targeting other parts of the molecule not defined by the HLAMatchmaker algorithm. Interestingly, in two other cases, the number of eplet MM was higher in the HLA molecule NOT targeted by the antibody (HR_dnDSA−) as compared to the specific one against which the antibody was directed (LR_dnDSA+), highlighting the importance of the type of molecular antigens more than their number, as hypothesized in previous studies (14).

### Impact of dnDSA on subsequent BPAR according to different HLA typing

Fifty-one patients (21%) developed BPAR, 35/51 (69%) TCMR and 17/51 (33%) ABMR. Twelve out of 17 (70.5%) patients with ABMR showed dnDSA, while five showed Banff compatible histological lesions with ABMR without detectable dnDSA neither with LR nor with complete HR HLA typing. Conversely, while no TCMR patients showed dnDSA at the time of rejection, 8 (35%) of them subsequently developed dnDSA over time.

**Figure 4:**
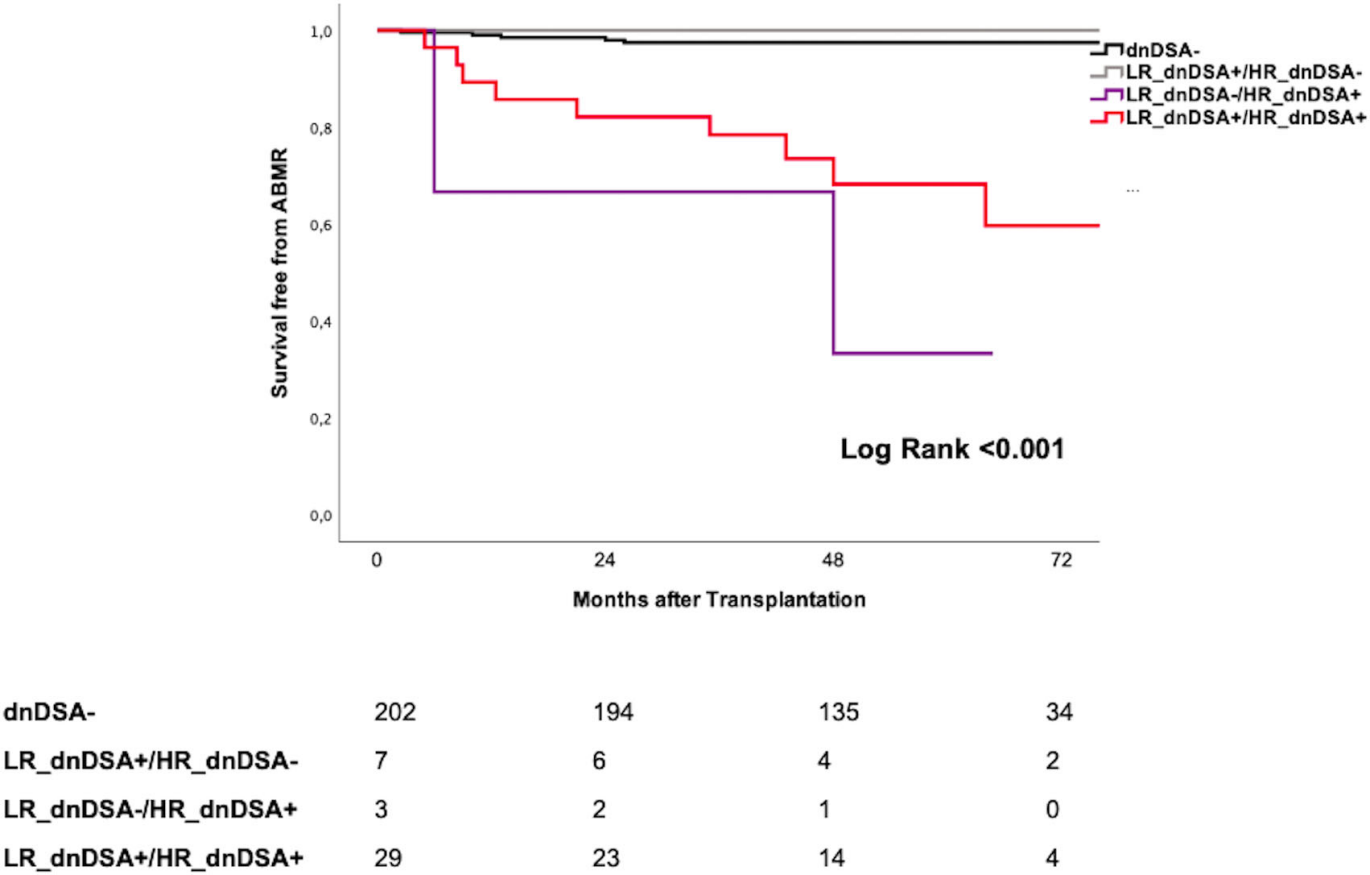
Kaplan Meyer Survival curves for ABMR according to different dnDSA identification. dnDSA, *de novo* donor -specific antibody; HR, high resolution; LR, low resolution; ABMR, antibody-mediated rejection.

When comparing the incidence of ABMR, LR_dnDSA +/HR_dnDSA+ patients displayed significantly higher cumulative rates as compared to dnDSA− patients and those only detected by LR HLA typing (LR_dnDSA+/HR_dnDSA−) (Log Rank < 0.001 global, Log Rank < 0.001 as compared to dnDSA−, Log Rank = 0.12 as compared to LR_dnDSA+/HR_dnDSA−) (**Figure 4**). Similarly, LR_dnDSA−/HR_dnDSA+ displayed higher risk as compared to LR_dnDSA+/HR_dnDSA− (Log Rank = 0.038). LR_dnDSA+/HR_dnDSA+ and LR_dnDSA-/HR_dnDSA+HR_dnDSA− displayed a higher risk of ABMR also when stratifying in class I and class II dnDSA (class I Log Rank < 0.001 for LR_dnDSA+/HR_dnDSA+ vs. dnDSA− and 0.21 vs. LR_dnDSA-/HR_dnDSA−, Log rank 0.66 for LR_dnDSA +/HR _dnDSA− vs. dnDSA−. There were no cases of LR_dnDSA −/HR_dnDSA+. class II Log Rank < 0.001 for LR_dnDSA +/HR_dnDSA+ vs. dnDSA− and 0.21 vs. LR_dnDSA +/HR_dnDSA− and 0.48 vs. LR_dnDSA −/HR_dnDSA+; Log rank 0.72 for LR_dnDSA+/HR_dnDSA− vs. dnDSA−; Log Rank < 0.001 for LR_dnDSA−/HR_dnDSA+ vs. dnDSA−).

At univariate logistic regression analysis including main clinical, demographic, and immunological variables, dnDSA defined as LR_dnDSA+, LR_dnDSA+/HR_dnDSA+ and LR_dnDSA −/HR_dnDSA+ were significant correlates of ABMR (**Table 4**).

**Table 4:** Univariate logistic regression analysis of main clinical, demographic, and immunological variables associated with ABMR.

Variables	OR (95% CI)	*p*
Recipient age (years)	0.98 (0.94–1.01)	0.20
Recipient gender (F)	0.31 (0.07–1.41)	0.13
ESRD cause (diabetes)	0.58 (0.05–6.73)	0.66
Recipient ethnicity (Caucasian)	0.78 (0.09–6.48)	0.82
Donor age (years)	0.99 (0.95–1.03)	0.53
Donor gender (F)	0.89 (0.31–2.54)	0.83
Transplant number (1)	0.65 (0.08–5.43)	0.69
Time on dialysis (months)	0.99 (0.98–1.01)	0.68
Type of transplantation (deceased donor)	1.21 (0.42–3.52)	0.72
Induction (rATG)	0.71 (0.19–2.64)	0.61
Maintenance CNI monotherapy	1.25 (0.38–4.09)	0.71
DGF	0.95 (0.26–3.51)	0.94
LR Typing HLA MM (1–6)	1.34 (0.86–2.08)	0.19
HR Typing HLA MM (1–12)	1.09 (0.87–1.38)	0.43
LR_dnDSA+	15.38 (4.88–48.52)	**<0.001**
LR_dnDSA+/HR_dnDSA+	21.79 (6.75–70.33)	**<0.001**
LR_dnDSA+/HR_dnDSA−	NA	
LR_dnDSA−/HR_dnDSA+	29.73 (2.55–346.89)	**0.007**

F, female; ESRD, end-stage renal disease; rATG, rabbit anti-thymoglobulin; CNI, calcineurin inhibitors; DGF, delayed graft function; LR, low resolution; HR, high resolution; MM, mismatch; dnDSA, *de novo* donor-specific antibody. Bold values highlights all p values <0.05. NA, Not applicable.

### Impact of dnDSA on the risk of graft loss according to different HLA typing

**Figure 5:**
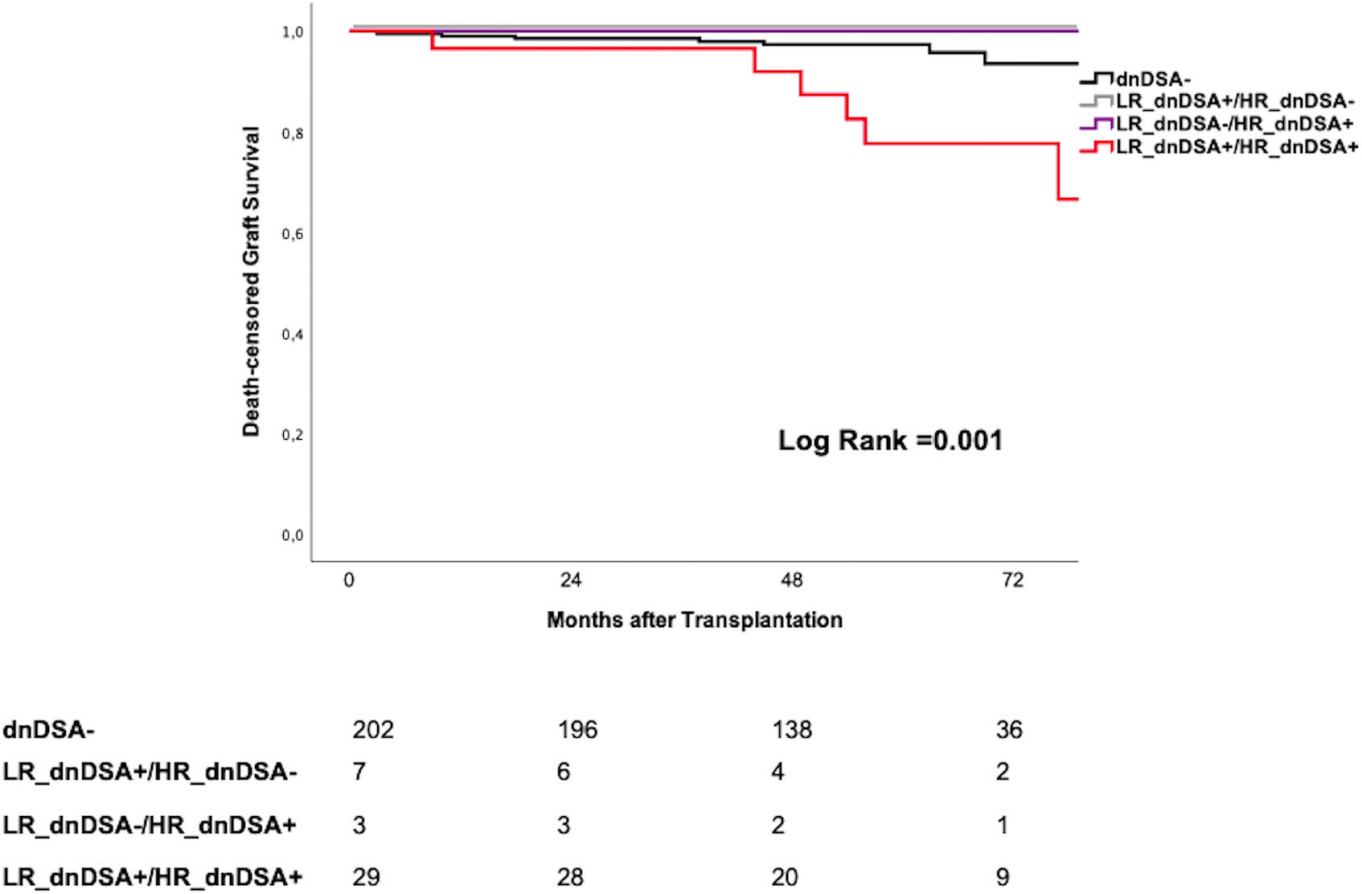
Kaplan Meyer Survival curves for death-censored graft loss according to different dnDSA identification. dnDSA, *de novo* donor -specific antibody; HR, high resolution; LR, low resolution.

Patients with LR_dnDSA+/HR_dnDSA+ showed significantly higher cumulative death-censored graft loss rates than all other groups (Log Rank < 0.001 global, Log Rank < 0.001 as compared to dnDSA−, Log Rank = 0.23 as compared to LR_dnDSA+/HR_dnDSA−, Log Rank = 0.39 as compared to LR_dnDSA−/HR_dnDSA+) (**Figure 5**). The same results were observed when breaking down dnDSA into class I and class II (data not shown).

At multivariate Cox regression analysis, transplant number (HR: 0.24, 95% CI = 0.07–0.84, *p* = 0.03), 12-month eGFR (HR: 0.94, 95% CI = 0.89–0.98, *p* = 0.004), previous BPAR (HR: 4.08, 95% CI = 1.23–13.52, *p* = 0.02), and both LR_dnDSA+ (HR: 3.51, 95% CI = 1.25–9.85, *p* = 0.017) and LR_dnDSA+/HR_dnDSA+ (HR: 4.09, 95% CI = 1.45–11.54, *p* = 0.008) independently predicted graft loss (**Table 5**). None of the patients with either LR_dnDSA +/HR_dnDSA− or LR_dnDSA−/HR_dnDSA+ lost their graft until last follow-up.

**Table 5:** Univariate and multivariate Cox regression analysis for death-censored graft loss.

	Univariate		Multivariate	
Variables	HR (95% CI)	*p*	HR (95% CI)	*p*
Recipient age (years)	1.01 (0.97–1.05)	0.45		
Recipient gender (F)	0.63 (0.20–1.95)	0.42		
ESRD cause (diabetes)	1.08 (0.21–5.47)	0.92		
Donor age (years)	1. 04 (1.002–1.08)	**0.04**	1.04 (0.99–1.09)	0.09
Donor gender (F)	0.61 (0.22–1.73)	0.36		
Transplant number (1)	0.17 (0.05–0.54)	**0.003**	0.24 (0.07–0.84)	**0.03**
Time on dialysis (months)	1.01 (1.002–1.02)	**0.009**	1.003 (0.99–1.01)	0.49
Type of transplantation (deceased donor)	1.41 (0.79–2.49)	0.24		
Induction (rATG)	1.31 (0.36–4.78)	0.68		
Maintenance CNI monotherapy	1.82 (0.41–8.10)	0.43		
LR Typing HLA MM (1–6)	1.08 (0.71–1.65)	0.72		
HR Typing HLA MM (1–12)	1.02 (0.82–1.27)	0.88		
DGF	3.32 (1.20–9.16)	**0.02**	1.97 (0.66–5.92)	0.23
12 months eGFR (ml/m/1.73 m^2^)	0.91 (0.88–0.95)	**<0.001**	0.94 (0.89–0.98)	**0.004**
BPAR	6.31 (2.33–17.11)	**<0.001**	4.08 (1.23–13.52)	**0.02**
*LR_dnDSA+	4.90 (1.82–13.19)	**0.002**	3.51 (1.25–9.85)	**0.017**
*LR_dnDSA+/HR_dnDSA+	4.90 (1.82–13.19)	**0.002**	4.09 (1.45–11.54)	**0.008**
LR_dnDSA+/HR_dnDSA−	NA			
LR_dnDSA−/HR_dnDSA+	NA			

*Independent models due to collinearity.

F, female; ESRD, end-stage renal disease; rATG, rabbit anti-thymoglobulin; CNI, calcineurin inhibitors; DGF, delayed graft function; LR, low resolution; HR, high resolution; MM, mismatch; eGFR, estimated glomerular filtration rate; BPAR, biopsy-proven acute rejection; dnDSA, *de novo* donor-specific antibody. Bold values highlights all p values <0.05. NA, Not applicable.

## Discussion

The use of complete HR HLA genotyping of donor/recipient pairs in kidney transplantation has been shown to have relevant implications in the pretransplant setting on the one hand to better define the degree of HLA matching and, on the other, to identify biologically impactful preformed DSA, especially those with anti-DQ specificity and when these antibodies are directed against HLA alleles not assessed by standard SSP/SSOP typing (6, 8, 15).

In this study, we rather focused on the impact of using complete two-field HR HLA genotyping on the identification of posttransplant immunologic events as compared to conventional one-field LR typing. Here, we show that those *de novo* anti-HLA antibodies that were confirmed to be specific against donor antigens by complete HR genotyping (LR_dnDSA+/HR_dnDSA+) displayed a deleterious effect on main allograft outcomes such as ABMR and poorer graft survival, whereas patients with anti-HLA antibodies that were not confirmed to be donor-specific (LR_dnDSA+/HR_dnDSA−) (20% of patients) did not show any significant risk of developing either ABMR or premature graft loss. Our study is the first report to show the clinical impact of using two-field resolution of HLA typing for a more accurate diagnosis of dnDSA arising after transplantation. It supports the recommendations made by distinct international working groups focusing on the pretransplant immune-risk stratification (3, 16), in which assessing donor/recipient with complete HLA typing at HR level seems to be highly warranted also for the posttransplant clinical follow-up and immunological risk stratification.

Notably, the most impactful difference between the two HLA typing approaches was the identification of patients developing dnDSA and the number of dnDSA specificities. Indeed, only 29/36 (80%) patients diagnosed to display dnDSA were confirmed when applying HR genotyping, and furthermore, three other patients were found to show dnDSA not identified by LR HLA typing. More importantly, when the number of dnDSA specificities was evaluated, these differences were even more relevant, since 49 dnDSA detected with LR changed to be only 34 (69%) with HR and 7 additional dnDSA were identified only by HR HLA typing. The main reasons for these discrepancies were the two-field resolution allele typing and the absence of DQA1 and DP typing using LR. Also, the absence of some HLA antigen representation in the SAB platform explained some incongruity, which highlights the limitation of using distinct SAB assays showing, albeit low (17), distinct sensitivity to detect DSA. These differences may have relevant clinical implication since, on the one hand, patients with a false-positive dnDSA determination may be exposed to invasive procedures such as protocol biopsies and even to antibody-depletion rescue therapies to eliminate alloantibodies and, on the other, patients with non-recognized circulating dnDSA with HR genotyping may undergo minimization strategies over time and be at higher risk of accelerated immune-mediated graft lesions and subsequent premature graft loss. Indeed, this was confirmed when the risk of ABMR and/or graft loss was shown to be only significant for those patients with confirmed dnDSA by complete HR HLA genotyping.

Correlation between HLA LR and HR MM was rather high but lacking important relevant alleles that may affect the generation of *de novo* alloimmune responses. Likewise, the degree of concordance of EpMM assessed by LR HLA typing introducing haplotype frequency imputation as compared to two-field complete HR was high. However, when assessing their predictive capacity of generating primary alloimmune activation, while patients developing dnDSA showed significantly higher EpMM, assessed by both technologies, especially class II molecules, a significantly higher association was clearly observed when using HR HLA genotyping. These data confirm previous observations on the importance of a correct imputation in the algorithm (7, 18). The fact that also EpMM calculated by LR typing and inferred two-field resolution associate to dnDSA appearance might be justified by high concordance at eplet level and is in accordance with the results that have been generated in the last decade using this methodology (19, 20). However, at the single-patient level, the estimation of the second field of resolution to calculate compatibility at the allele and eplet level, as well as to diagnose dnDSA, seems to run into some relevant pitfalls and must be discouraged especially if EpMM are used to classify patients in clinical trials or for clinical decision-making.

Importantly, NGS technology for HLA genotyping provides HLA data up to the four‐field level (including information on synonymous substitutions and changes in non-coding regions). In our study, similar to most recent reports in this field, we focused on the accurate characterization of two-field (four digits) typing giving information on the complete protein elements of the HLA molecule. Nonetheless, the clinical application of the knowledge of the third/fourth field needs to be further explored in solid-organ transplantation (21).

In the era of modern medicine, the individualization of immunosuppressive treatment and diagnostic procedures is highly recommended and different consortia are performing clinical interventional trials in this direction (EUtrain: NCT03652402; Outsmart: EudraCT 2012–004308-36; BIOIMMUN: EudraCT 2017-002293-39). However, any implementation in clinical practice of biomarkers used to guide medical decisions should rely on the highest technological standards with the best accuracy when defining high- or low-risk patients. According to our results, the use of HR complete HLA genotyping significantly improves the correct identification of DSA, both prior to and after kidney transplantation to precisely rule out the presence of preformed and *de novo* serological anti-donor immunity, respectively, which may ultimately affect graft outcomes.

## Data availability statement

The raw data supporting the conclusions of this article will be made available by the authors, without undue reservation.

## Ethics statement

This study was reviewed and approved by Bellvitge University Hospital. The patients/participants provided their written informed consent to participate in this study.

## Author contributions

OB designed the study. MM, FB, PR, OV, MG, EM, AS, LH, PH, CG-G, SB, JG and OB had enrolled patients in the study. MM, AP, FB, PR, OV, MG, EP, EM, JZ, AS, NL, LH, PH, CGG and SB collected clinical and immunological data. MM, AP, EC, EP, AT, LD, JG and OB participated in data analysis. MM, AP, EC and OB wrote the manuscript. All authors contributed to the article and approved the submitted version.

## Funding

This work was supported by the Biomarker-Driven Immunosuppression Minimization (BIO-DRIM) Consortium (EU FP7-health, grant agreement number 305147; FP7/2012-2017) and by the Instituto de Salud Carlos III (ISCIII) (grant numbers ICI14/00242, PI16/01321, and PI19/01710), co-funded by European Regional Development Fund (ERDF), a way to build Europe. Also, this work was partly supported by the SLT002/16/00183 grant, from the Department of Health of the Generalitat de Catalunya by the call “Acció instrumental de programes de recerca orientats en l’àmbit de la recerca i la innovació en salut”.

## Acknowledgments

We acknowledge the assistance of our lab technicians for careful management of all biological samples. Samples from patients included in this study were provided by the Biobank HUB-ICO-IDIBELL (PT17/0015/0024), integrated in the Spanish Biobank Network, and they were processed following standard operating procedures with the appropriate approval of the Ethics and Scientific Committees.

## Conflict of interest

The authors declare that the research was conducted in the absence of any commercial or financial relationships that could be construed as a potential conflict of interest.

## Publisher’s note

All claims expressed in this article are solely those of the authors and do not necessarily represent those of their affiliated organizations, or those of the publisher, the editors and the reviewers. Any product that may be evaluated in this article, or claim that may be made by its manufacturer, is not guaranteed or endorsed by the publisher.
