# Supplementary material for: On the clinical relevance of using complete high-resolution HLA typing for an accurate interpretation of posttransplant immune-mediated graft outcomes
Source: Front Immunol. 2022 Sep 29;13:924825. doi: 10.3389/fimmu.2022.924825 (PMC9559221; doi:10.3389/fimmu.2022.924825)

Supplementary Material

# Supplementary Tables

**Supplemental Table 1** Mean number of HLA Eplet mismatches calculated from LR HLA typing (A, B, DRB1+ estimation DQB1 typing) and complete HR HLA typing (A, B, C, DRB1, DQA/B, DPB1)

| HLA Eplet mismatches | LR HLA typing LR_EpMM | HR HLA typing  HR_EpMM |
| --- | --- | --- |
| Class I | 11.87 ± 5.39 | 13.22 ± 5.93 |
| Ab Verified class I | 7.30 ± 3.41 | 8.22 ± 3.84 |
| Class II | 14.63 ± 9.95 | 20.94 ± 12.44 |
| Ab Verified class II | 5.51 ± 3.97 | 8.31 ± 5.15 |

**Abbreviations:** EpMM: eplet mismatches; LR: low-resolution; HR: high-resolution; Ab: antibody.

## 2 Supplementary Figures

**Supplementary Figure 1.** Correlation between Class I (A) and Class II (B) eplet MM calculated either from LR or complete HR HLA Typing: Figure S1C shows the correlation when HR DQ and DPB1 loci are included as well as DQB1 estimation from LR DRB1 HLA typing.

**Abbreviations:** EpMM: eplet mismatches; LR: low-resolution; HR: high-resolution; Ab: antibody.

**Supplemental Figure 2** Association between Class II LR_EpMM (A) and Class II HR_EpMM (B) with anti-class II dnDSA formation.

Association between DRB1 LR_EpMM (C) and DRB1 HR_EpMM (D) with anti-DRB1 dnDSA formation.

Association between DQB1 LR_EpMM (E) and DQ HR_EpMM (F) with anti-DQ dnDSA formation.


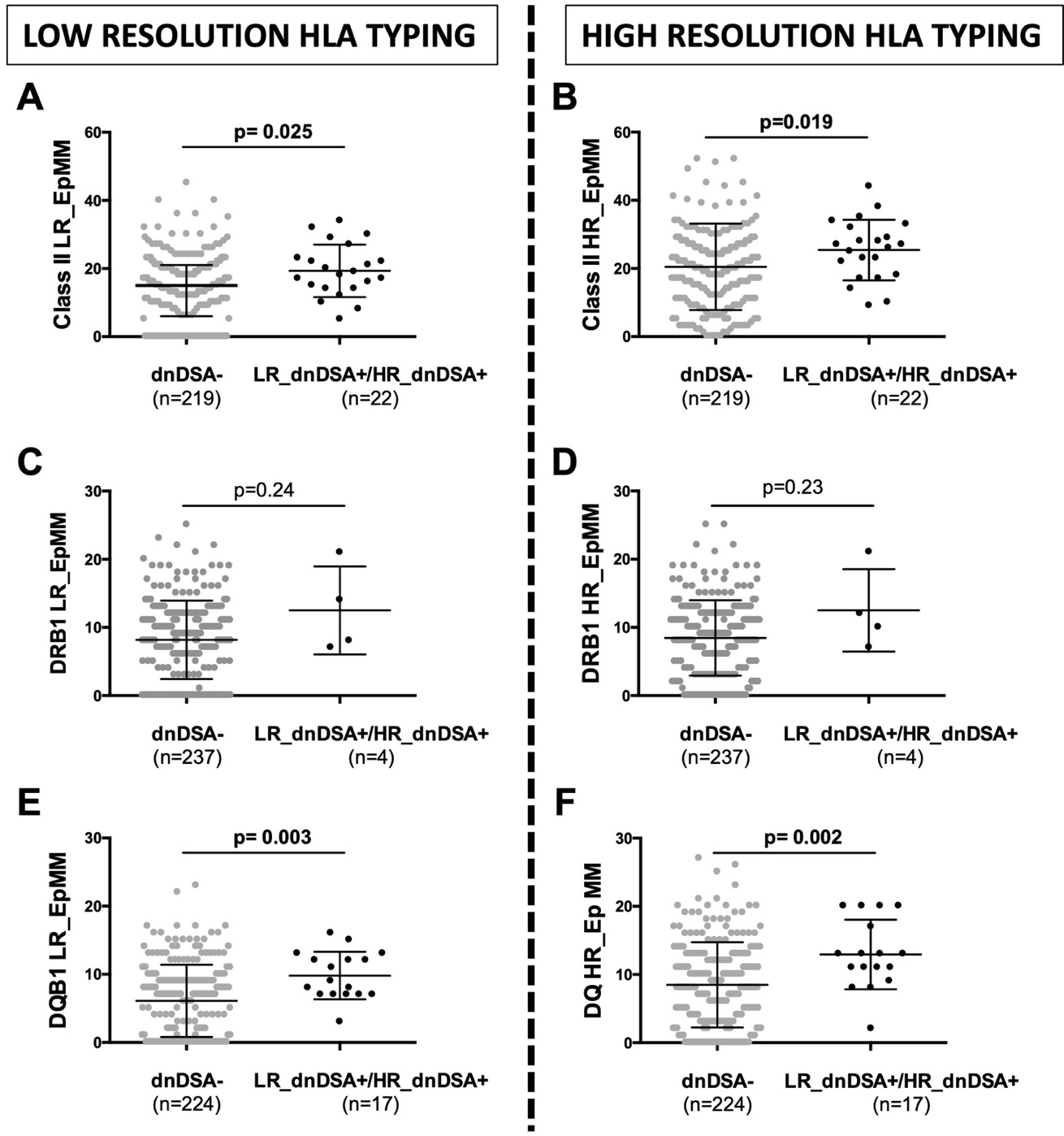

Supplement: Supplementary file 1 [file DataSheet_1.docx]
